# Effects of Blood-Glucose Lowering Therapies on Body Composition and Muscle Outcomes in Type 2 Diabetes: A Narrative Review

**DOI:** 10.3390/medicina61081399

**Published:** 2025-08-01

**Authors:** Ioana Bujdei-Tebeică, Doina Andrada Mihai, Anca Mihaela Pantea-Stoian, Simona Diana Ștefan, Claudiu Stoicescu, Cristian Serafinceanu

**Affiliations:** 1 Department of Diabetes, Nutrition and Metabolic Diseases, University of Medicine and Pharmacy “Carol Davila”, 030167 Bucharest, Romania; 2 National Institute of Diabetes, Nutrition and Metabolic Diseases “Prof. N. Paulescu”, 030167 Bucharest, Romania; 3 Department of Cardiology and Cardiovascular Surgery, University of Medicine and Pharmacy “Carol Davila”, 030167 Bucharest, Romania; 4 Department of Cardiology, University Emergency Hospital of Bucharest, 050098 Bucharest, Romania

**Keywords:** type 2 diabetes, body composition, sarcopenia, insulin, GLP-1 receptor agonist, SGLT2 inhibitors, tirzepatide, muscle mass

## Abstract

*Background and Objectives*: The management of type 2 diabetes (T2D) extends beyond glycemic control, requiring a more global strategy that includes optimization of body composition, even more so in the context of sarcopenia and visceral adiposity, as they contribute to poor outcomes. Past reviews have typically been focused on weight reduction or glycemic effectiveness, with limited inclusion of new therapies’ effects on muscle and fat distribution. In addition, the emergence of incretin-based therapies and dual agonists such as tirzepatide requires an updated synthesis of their impacts on body composition. This review attempts to bridge the gap by taking a systematic approach to how current blood-glucose lowering therapies affect lean body mass, fat mass, and the risk of sarcopenia in T2D patients. *Materials and Methods*: Between January 2015 and March 2025, we conducted a narrative review by searching the PubMed, Scopus, and Web of Science databases for English-language articles. The keywords were combinations of the following: “type 2 diabetes,” “lean body mass,” “fat mass,” “body composition,” “sarcopenia,” “GLP-1 receptor agonists,” “SGLT2 inhibitors,” “tirzepatide,” and “antidiabetic pharmacotherapy.” Reference lists were searched manually as well. The highest precedence was assigned to studies that aimed at adult type 2 diabetic subjects and reported body composition results. Inclusion criteria for studies were: (1) type 2 diabetic mellitus adult patients and (2) reporting measures of body composition (e.g., lean body mass, fat mass, or muscle function). We prioritized randomized controlled trials and large observational studies and excluded mixed diabetic populations, non-pharmacological interventions only, and poor reporting of body composition. *Results*: Metformin was widely found to be weight-neutral with minimal effects on muscle mass. Insulin therapy, being an anabolic hormone, often leads to fat mass accumulation and increases the risk of sarcopenic obesity. Incretin-based therapies induced substantial weight loss, mostly from fat mass. Notable results were observed in studies with tirzepatide, demonstrating superior reduction not only in fat mass, but also in visceral fat. Sodium-glucose cotransporter 2 inhibitors (SGLT2 inhibitors) promote fat loss but are associated with a small yet significant decrease in lean muscle mass. *Conclusions*: Blood-glucose lowering therapies demonstrated clinically relevant effects on body composition. Treatment should be personalized, balancing glycemic control, cardiovascular, and renal benefits, together with optimal impact on muscle mass along with glycemic, cardiovascular, and renal benefits.

## 1. Introduction

T2D is one of the major global health issues, affecting approximately 589 million. Projections suggest that by 2050, nearly 853 million individuals, diagnosed or undiagnosed, may be affected worldwide. Such a huge rise calls for urgent and immediate public health intervention. With an estimated 11.1% of all adults aged 20 to 79 years having diabetes, T2D stands as a great medical and economic concern [1].

Historically, the management of T2D centered mainly on achieving glycemic control to avoid microvascular and macrovascular complications [2]. However, there is a growing understanding that T2D management should extend beyond blood-glucose control to achieve optimal body composition, as it is naturally linked with overweight and obesity [3]. This is driven by increasing evidence that adverse changes in body composition in T2D, such as loss of muscle and its functional impairment, are associated with increased risks for functional impairment, frailty, disability, falls, fracture, hospitalization, cardiovascular illness, and mortality, all of which negatively affect the health-related quality of such patients [4].

These complications include sarcopenia and sarcopenic obesity (SO). Sarcopenia, once defined as chronic age-related loss of skeletal muscle mass (SMM), strength, and/or function, is increasingly regarded as a T2D-accelerated complication [5]. SO, or the presence of both low muscle mass/function and excess adiposity, is a very unfavorable phenotype that indicates a combined augmentation of metabolic risk, disability, and mortality over each alone [6]. The diagnosis of such disorders remains challenging due to the varying definitions and criteria proposed by various working groups (e.g., EWGSOP, AWGS, IWGS, FNIH, SDOC) employing different sets of low muscle mass, low muscle strength, and impaired physical performance measures [2]. This lack of consensus changes the prevalence estimates and comparative research.

The Mediterranean diet, plant-based and high-fiber diets, and particular eating patterns like intermittent fasting are just a few of the interventions that are suggested to improve insulin sensitivity and lower the incidence of T2D, according to a recent expert opinion [7].

T2D not only increases fat mass but also accelerates the decline of skeletal muscle mass, strength, and function—signs of sarcopenia. This relationship is supported by cohort studies showing greater muscle loss and higher sarcopenia risk in adults with T2D up to 1.5- to 3-fold increased risk than adults without diabetes [8]. Reduced muscle mass may impair glucose disposal, contributing to worsening glycemic control [9]. The underlying mechanisms include insulin resistance, chronic inflammation, oxidative stress, mitochondrial dysfunction, and myosteatosis [10,11].

Newer therapies for T2D have brought forward glucose-lowering agents that address weight and cardiometabolic risk reduction together. However, unwanted muscle loss became an issue, especially in elderly patients or patients with sarcopenia or SO. Given these shortcomings, there is a requirement for a targeted synthesis of recent evidence to explain the extent and clinical relevance of muscle changes resulting from blood-glucose lowering therapies and to guide tactics which will avoid lean body mass (LBM) loss while retaining overall therapeutic benefit.

This review aimed to investigate the effects of primary antidiabetic treatments on the body composition of patients with T2D using data derived from randomized controlled trials (RCTs), meta-analyses, and observational studies conducted between 2015 and 2025. It includes metformin (biguanides), insulin, GLP-1 receptor agonists, SGLT2 inhibitors, and the dual GLP-1/GIP agonist tirzepatide. Metformin was chosen as the sole biguanide representative due to its modern widespread use and data availability. The remaining pharmacological groups, such as glitazones and sulfonylureas, were eliminated due to decreased clinical use or inconsistent reporting of body composition outcomes.

## 2. Metformin

Given its widespread use and neutral wight profile, metformin’s effects on body composition remain of interest [6].

### 2.1. Effects on Body Weight and Fat Mass

Compared to older antidiabetic medications (e.g., sulfonylureas, thiazolidinediones, insulin), metformin is primarily associated with minimal weight loss or, more precisely, weight neutrality [9]. Meta-analysis evidence reported in 2020 supports a statistically significant but modest reduction of body mass index (BMI) typically by 1 kg/m^2^ relative to placebo or comparator treatment [10]. A more recent publication in 2021 using more than one method of body composition measurement established that the reduction in fat mass is primarily the cause of this effect on weight [9]. Some evidence shows preferential visceral adipose tissue (VAT) reduction, a metabolically unfavorable depot of fat [11], but not a result shared by all studies.

The Diabetes Prevention Program (DPP) also reported that metformin reduced body weight and subcutaneous fat, but its capacity to prevent diabetes onset was unrelated to these adiposity changes [12]. Fat reduction mechanisms induced by metformin may comprise reduced appetite, lowered fat absorption from the gut, increased fat oxidation, and possibly adipocyte differentiation and inflammation regulation [13].

### 2.2. Impact on Lean Mass and Muscle

The effects of metformin on LBM and muscle health are complex and somewhat paradoxical. A small study reported increases in LBM and decreases in fat mass. Moreover, long-term data from the Osteoporotic Fractures in Men (MrOS) study showed some valuable data: in diabetic men aged 65 and older, treatment with insulin sensitizers (mostly metformin, sometimes alongside thiazolidinediones) was associated with significantly slower loss in both appendicular and total lean mass as they aged, compared to untreated diabetic men and even healthy men of the same age [14]. This finding is in line with others that suggest that metformin could help lower the risk of sarcopenia in older adults with T2D [15].

However, the picture is more complex. For example, the large, randomized MASTERS trial adds nuance. In that study, healthy adults aged 65 and older received either metformin (1700 mg daily) or placebo while undergoing 14 weeks of supervised progressive resistance training [16]. Surprisingly, the group treated with metformin experienced significantly smaller gains in both LBM and thigh muscle mass compared to the placebo. Specifically, the placebo group had greater increases in LBM (*p* = 0.003) and thigh muscle mass (*p* < 0.001). Computed tomography also confirmed larger improvements in thigh muscle area (*p* = 0.005) and density (*p* = 0.020) among the placebo group. While strength increased in both groups, there was a noticeable trend toward smaller strength gains in patients treated with metformin, particularly in knee extension strength and power. These findings are consistent with a previous, smaller adult prediabetes study that similarly showed metformin blunted exercise-induced lean mass gains during concurrent aerobic and resistance training [16].

In contrast, some network meta-analyses pooling across various T2D trials reported no difference between placebo and metformin in fat-free mass (FFM) change. On the other hand, agents like semaglutide and certain SGLT2 inhibitors did lower FFM significantly from placebo [9].

### 2.3. Impact on Sarcopenia Risk

The contradictory evidence for lean mass makes it difficult to assess the direct impact of metformin on sarcopenia risk. Observational accounts in T2D cohorts all report a protective association, where metformin users have a lower rate or incidence of sarcopenia compared with non-users or users of alternative treatments [16,17,18,19]. These observational accounts often adjust for confounders, but residual confounding or indication bias cannot be excluded completely. Metformin has long been recognized for its broad metabolic effects, particularly its ability to improve glycemic control, reduce systemic inflammation, and enhance insulin sensitivity—mechanisms attributed mainly to its activation of AMP-activated protein kinase (AMPK) [18,19]. This all seems promising for muscle health at first glance. Yet, evidence from the MASTERS trial complicates the narrative. While metformin may dampen inflammation-driven muscle breakdown in individuals with type 2 diabetes, it also appears capable of blunting the anabolic, or muscle-building, response to resistance training. In other words, the drug might simultaneously help preserve muscle in pathological conditions but hinder gains when exercise is introduced. The implications are nuanced and context-dependent—factors such as diabetes, the specific muscle outcome of interest (maintenance versus growth), and the introduction of exercise all shape metformin’s impact on muscle tissue [16].

In conclusion, while metformin remains a cornerstone of T2D management and provides significant benefits in weight and fat mass reduction compared to previous agents, its net effects on muscle health are complex and highly dependent on individual context. Although observational data in T2D patients suggest a protective relationship against sarcopenia, possibly due to improved metabolic control and anti-inflammatory effects, strict RCT data in healthy older individuals show that it can reduce the hypertrophic muscle effect of resistance training, possibly by inhibiting mTORC1 [16]. This means that, while it is beneficial for general body composition management in the majority of T2D patients, it should be administered with caution in individuals, especially older adults, who are actively involved in resistance training routines to optimize muscle development. More study is needed to account for these context-dependent behaviors and improve combined exercise and metformin outcomes.

## 3. Insulin Therapy

Insulin, initially isolated over a century ago, remains a critical therapy for the management of hyperglycemia in patients with longstanding T2D or known β-cell failure [18]. While insulin is highly effective at lowering glucose, its effects on fat accumulation and lean mass preservation in T2D pose significant clinical challenges [20].

### 3.1. Effects on Body Weight and Fat Mass

A consistent and well-documented effect of initiating or intensifying insulin therapy in T2D is weight gain [6]. This effect was noted in groundbreaking studies, such as the UKPDS, and continues to be a source of concern for patients as well as for clinicians, potentially acting as a disincentive to the premature intensification of treatment [21]. The mechanisms behind insulin-induced weight gain are reduced glycosuria (retention of calories previously lost in urine), a potential increase in appetite, and direct anabolic effects of insulin, promoting energy storage [22]. Body composition studies indicate that such weight gain typically comprises fat mass and fat-free mass increments [20]. But the relative contribution from each compartment is debated. Disproportionate FM gain, particularly in the trunk, has been implicated in certain reports and may foster visceral adiposity [20]. Such fat distribution is metabolically unfavorable since visceral fat is strongly linked with insulin resistance, inflammation, and cardiovascular disease [23]. Of note, the initial weight gain with initiating insulin may be secondary to water retention from increased hydration of lean tissues as hyperglycemia resolves [24].

### 3.2. Effects on Lean Mass and Muscle

With the powerful anabolic effects of insulin, it only makes sense that it would be a buffer against muscle loss. Insulin signaling activates muscle amino acid and glucose uptake, initiates muscle protein synthesis (MPS) by Akt and mTORC1 pathways and suppresses muscle protein breakdown (MPB) [25]. Arteriovenous balance techniques in limbs replicate these data to demonstrate that infusion of insulin is accompanied by elevated net amino acid uptake into muscle, a composite anabolic response [26]. However, the net effect of activating MPS or suppressing MPB in humans would appear to be a function of ambient amino acid concentration and muscle blood flow, which can also be controlled by insulin [26].

In the specific context of T2D, insulin’s beneficial actions on muscle can be disrupted. Insulin resistance, which is the basic defect in T2D, will inherently dampen the muscle’s response to insulin signaling [25]. Beyond that, pathologies like endothelial dysfunction and microvascular damage may undermine insulin’s capacity to augment blood flow and nutrient delivery to the muscle [27]. Observational and longitudinal research provides a mixed picture. While T2D itself is associated with accelerated muscle loss, some evidence suggests that exogenous insulin therapy might help mitigate this decline. For instance, Tanaka et al. found that reduced endogenous insulin secretion was a separate risk factor for sarcopenia in men with T2D, indicating an insulin protective effect [28]. Another cohort study (KORA-Age) reported that older adults with T2D on insulin treatment preserved their skeletal muscle index at 3 years compared with oral agent alone therapy, although preservation did not extend to muscle function (measured by grip strength or a Timed Up and Go test) [29]. A study also reports associations between insulin treatment and an attenuated decline in lower-extremity muscle mass or strength [26]. Conversely, the very need for insulin treatment typically mirrors more advanced diabetes duration and perhaps more severe underlying metabolic disturbances, which are independent predictors of wasting muscle [30].

### 3.3. Impact on Sarcopenia Risk

The net effect of insulin therapy on T2D sarcopenia risk is complex and adverse compared to newer agents, despite its anabolic properties. While some studies report that insulin can preserve muscle mass or restrict strength decline in certain muscle compartments [20], this potential benefit must be weighed against significant liabilities. The consistent correlation with fat gain and potentially harmful visceral fat deposition [20], can also aggravate overall metabolic status and potentially culminate in SO. A cross-sectional study identified a low prevalence of definite sarcopenia (1.6%) in elderly subjects with T2D on insulin, according to EWGSOP2 criteria, which may confirm an anabolic effect [31]. However, this finding contrasts with the often-reported higher prevalence of sarcopenia in patients with T2D as a whole and requires confirmation through longitudinal studies assessing functional outcomes. The demand for insulin typically follows the duration and severity of illness, which are independently related to the risk of sarcopenia [30]. Therefore, it is not straightforward to differentiate the effect of treatment with insulin from patient factors.

Therefore, insulin treatment of T2D entails a significant body composition trade-off. Its anabolic character would theoretically be beneficial to preserving muscle mass over the accelerated loss of T2D. But this would frequently be counterbalanced by high, generally adverse (visceral) fat gains and an inability to maintain improvements in muscle quality or function. The inherently complex interaction between insulin’s direct effects, the inherent severity of T2D in insulin-treated individuals, and the influence of concurrent fat accretion makes its net contribution to preventing or treating sarcopenia questionable compared to treatments that produce fat loss with the additional potential of having a more neutral or even a positive influence upon the fat-to-lean mass ratio.

## 4. Sodium-Glucose Cotransporter 2 Inhibitors (SGLT2 Inhibitors)

SGLT2 inhibitors like empagliflozin, canagliflozin, and dapagliflozin are another large class of new antidiabetic agents that have changed the course of T2D management. Their administration is also associated with weight loss, and thus research has been conducted on their effects on various parts of the body [32].

### 4.1. Effects on Body Weight and Fat Mass

SGLT2 inhibitors cause steady and modest weight loss, frequently averaging 2–3 kg over 6–12 months in clinical trials [33,34,35]. Robust meta-analyses demonstrate significant decreases in body weight, BMI, and waist circumference compared with placebo or other active comparators (excluding GLP-1 RAs) [36]. Compositional analyses by DXA, BIA, CT, or MRI to assess body composition show that a reduction in fat mass is the major driver of this weight loss [37]. Pooling analyses demonstrate significant decreases in total fat mass, percentage body fat, VAT, and SAT [38]. In the Pioneer-2 study, empagliflozin induced modest weight loss, primarily through fat mass reduction, with little impact on LBM [38,39].

### 4.2. Effects on Lean Mass and Muscle

Various systematic reviews and meta-analyses of RCTs consistently identify that SGLT2 inhibitors therapy is associated with a statistically significant reduction in LBM and/or SMM compared to control groups [40]. The magnitude of the reduction is usually modest, with weighted mean differences typically around −0.7 kg for LBM and up to approximately −1.0 kg for SMM in some analyses [38]. This contrasts with metformin, where meta-analyses did not find a significant reduction in FFM compared to placebo [9]. The proportion of total weight loss attributed to LBM with SGLT2 inhibitors appears to be in the 20–50% range [41]. In certain studies, this proportion is estimated at around 20–30% [9].

A potential impact factor here is the impact of fluid loss on LBM measurements. SGLT2 inhibitors induce osmotic diuresis, particularly during the initiation of therapy, resulting in initial weight reduction and affecting hydration status [32]. Trials using bioimpedance spectroscopy, which can differentiate between fluid compartments, show that initial weight loss primarily reflects a reduction in extracellular fluid. Later, actual LBM tends to stabilize or show less significant losses [42,43,44]. Meta-analyses consistently show marked reductions in total body water with SGLT2 inhibitors treatment [40]. Even after adjusting for fluid shift, numerous meta-analyses using different measurement methods (including DXA) still report reduced solid lean tissue or muscle mass, suggesting the loss is beyond mere dehydration [4].

### 4.3. Impact on Muscle Function and the Risk of Sarcopenia

The consistent finding of LBM/SMM decline has raised significant concern regarding whether SGLT2 inhibitors can increase the risk of sarcopenia or exacerbate muscle dysfunction, particularly in at-risk groups like the elderly or those with underlying frailty [43]. Several reviews and meta-analyses themselves explicitly raise this potential risk and advise caution [38]. Moreover, one meta-analysis specifically addressing sarcopenia-related outcomes reported that SGLT2 inhibitors therapy was associated with statistically lower muscle strength compared to controls, in addition to the LBM/SMM declines [45].

Despite these concerns based on body composition data and isolated reports, large CVOTs involving tens of thousands of patients overall have not found sarcopenia, frailty, or serious adverse events related to muscles to be a top-line safety issue, although these outcomes were not typically primary or systematically assessed endpoints. The safety profile in the elderly tends to be concluded as follows: while there is a need for caution in terms of risk of volume depletion or infection, the benefit-risk profile, in general, is favorable for large cardiorenal outcomes and comparable to that of younger individuals [46]. Yet, the discrepancy between uniform LBM loss demonstrated in meta-analyses and the lack of any obvious functional impairment documented in large CVOTs serves to emphasize the necessity of investigations specifically evaluating muscle function and physical performance during SGLT2 inhibitors treatment.

Despite this, SGLT2 inhibitors offer substantial cardiorenal benefits and reduce fat via their unique insulin-independent glycosuric mechanism (daily caloric loss of approximately 280–320 kcal) [46]. The consistent outcome in most meta-analyses of the resultant loss of LBM and/or SMM is an extremely relevant clinical warning regarding the risk of sarcopenia. While the long-term implications for muscle function are not yet clear, due to limited trial endpoints, the present evidence calls for clinical awareness. Clinicians should proactively monitor muscle function and initiate preventative action, in the form of resistance exercise and adequate provision of protein.

## 5. Glucagon-like Peptide-1 Receptor Agonists (GLP-1 RAs)

GLP-1 RAs represent a major therapeutic development in the management of T2D, extending beyond glycemic control, weight management, and cardiovascular risk reduction [47]. This class includes liraglutide, semaglutide, dulaglutide, exenatide, etc. They are incretin mimetics, enhancing the physiological effects of endogenous GLP-1 [41]. Its effective and notable weight loss effects raised considerable concern about its impact on muscle mass and function.

### 5.1. Effects on Body Weight and Fat Mass

GLP-1 RAs are associated with a predictable and meaningful effect on weight loss in subjects with T2D and/or obesity [48]. The loss of weight is usually dose-dependent and significant with more recent therapies like semaglutide [49]. Weight reduction is primarily fat mass-driven, as shown by using several different methods for measuring body composition (DXA, CT, and MRI) [50]. Studies show reductions in total body fat, percentage body fat, VAT, and SAT [51]. Reducing VAT is particularly beneficial, since VAT is strongly linked to metabolic dysfunction. Some show equal absolute drops in VAT and SAT, while others record preferential VAT loss or favorable redistribution [52].

### 5.2. Impact on Lean Mass and Muscle

The substantial weight loss associated with GLP-1 RAs inherently raises concerns about the loss of LBM or SMM. This is a crucial consideration, since LBM plays a key role in metabolism, strength, function, and maintenance of metabolic rate [41].

The evidence about the percentage of LBM or muscle mass lost with GLP-1 RA treatment is mixed. Meta-analyses that pool outcomes of RCTs also typically show a statistically significant reduction in absolute LBM or FFM compared to controls [8]. However, one meta-analysis focused on T2D patients found that the average reduction did not reach statistical significance. In non-diabetic subjects treated with GLP-1 RA for obesity, there is typically an LBM loss, although less than fat mass loss [52,53].

A commonly used measure is the proportion of total weight loss that is attributed to LBM. Several reviews found that this proportion usually ranges from 20% to 50%. A few single-trial reports, such as substudies from the STEP 1 and SUSTAIN 8 trials with semaglutide, have presented even higher ratios. It started at 40% or sometimes exceeding 60% (for liraglutide in one overview, though this might be an outlier) [54]. Importantly, this range is generally comparable to those resulting from extreme weight loss induced by other treatments, for instance, low-calorie diets or bariatric surgery [41]. This similarity suggests that LBM loss is at least in part a normal physiological response to severe weight loss rather than a specific adverse effect of GLP-1 RAs.

Despite these losses, overall body composition improved. Many analyses reported an increase in the ratio of LBM to body weight, showing a greater reduction in fat mass [55]. Moreover, newer analyses involving MRI data suggest that the changes in muscle volume with GLP-1 RA treatment can be considered adaptive (the reduction is proportionate to the body weight loss and the reduced load on muscles). These changes do not seem to alter muscle function or integrity [55]. Maintenance of FFM and SMM has even been observed at 26 weeks in a real-world clinical practice with oral semaglutide [56].

### 5.3. Impact on Muscle Function and Sarcopenia Risk

The direct measurement of muscle strength, physical function, and the incidence of sarcopenia has not been a main outcome in the majority of large GLP-1 RA cardiovascular or weight loss studies [53]. As a result, information on functional outcomes is limited. Measured losses of LBM deserve serious consideration as adverse functional outcomes in high-risk populations such as the elderly or those with early frailty [57]. A Japanese observational study reported an increased fall risk in T2D patients being treated with GLP-1 RA. This raised the possible correlation with frailty or sarcopenia but did not prove causality [58]. Yet other studies involving patients with sarcopenia in GLP-1 RA trials have been conducted. However, there were no muscle-specific adverse results observed, and these were not formally evaluated [47]. Consensus in the present day agrees that LBM loss is a proven and clinically significant phenomenon that requires treatment. However, no large trial has convincingly linked GLP-1 RA therapy per se with an increased risk of clinically defined sarcopenia or enduring functional impairment [59]. However, the lack of specifically dedicated functional data represents a major gap. This calls for precautions, monitoring, and active management (e.g., exercise, diet), particularly in high-risk individuals.

The development of GLP-1 RAs represents a significant step forward in the management of T2D and obesity, offering substantial benefits in reducing fat and improving metabolic well-being. However, the persistent observations of concomitant LBM loss, even when often proportional to fat loss and perhaps adaptive in some cases, cannot be overlooked. Such observations demand a paradigm shift in the clinic. When celebrating the attainment of weight loss in fat, clinicians should also attempt parallel interventions to preserve muscle mass and, more importantly, muscle function under GLP-1 RA therapy. This involves careful monitoring of body composition beyond simple weight tracking and the application of adjunctive modalities, such as structured resistance exercise and sufficient protein intake, particularly in the elderly or those at baseline risk of sarcopenia, to optimize long-term health and functional gain.

## 6. Tirzepatide (Dual GIP/GLP-1 RA)

Tirzepatide is a new therapeutic modality, the first clinically approved dual agonist of the glucose-dependent insulinotropic polypeptide (GIP) and GLP-1 receptors [60]. It is dosed once weekly by subcutaneous injection. It produces unprecedented efficacy in glycemic control and in causing significant weight loss, resulting in its approval for both T2D and chronic weight management [61]. Its significant effect on body weight warrants scrutiny of its impact on body composition, specifically lean mass preservation.

### 6.1. Effects on Body Weight and Fat Mass

Clinical trial programs (SURPASS in T2D, SURMOUNT in obesity) have consistently demonstrated that tirzepatide induces much more pronounced weight loss than placebo and has superior efficacy compared to selective GLP-1 RAs like semaglutide (1 mg dose). In T2D patients, weight losses of up to 15% have been reported, and in non-diabetic patients with obesity, weight loss amounted to approximately 21–22% with higher doses (10 mg, 15 mg) over 72 weeks [61,62]. A dramatic reduction in fat mass is the main contributor to this extensive weight loss [59]. In a DXA substudy of the SURMOUNT-1 trial (non-T2D subjects), the overall mean reduction in total fat mass was approximately 34% with tirzepatide (pooled doses) compared to approximately 8% with placebo over 72 weeks [63]. Furthermore, tirzepatide treatment is associated with considerable reductions in metabolically unhealthy fat depots like VAT and liver fat content, often with superiority over comparator medications like insulin degludec [64]. An MRI substudy of the SURPASS-3 trial (T2D patients) demonstrated superior tirzepatide-induced reductions in VAT and liver fat for all tirzepatide doses compared to insulin degludec [65]. Exploratory analyses even suggest a shift toward a more favorable pattern of fat distribution, with a greater relative reduction in VAT compared to abdominal subcutaneous adipose tissue (ASAT) [65].

### 6.2. Impact on Muscle and Lean Mass

Based on the degree of total weight loss caused by tirzepatide, a comparable decrease in absolute LBM is predictable and is seen [60]. Within the SURMOUNT-1 DXA substudy, subjects treated with tirzepatide lost on average around 11% of LBM from baseline compared to roughly 2.6% for those in the placebo group. Nonetheless, the question concerns what percentage of the weight loss comes from lean tissue as opposed to fat tissue. In the SURMOUNT-1 substudy, fat mass loss was indeed approximately threefold greater than lean mass loss [63]. This suggests that LBM loss contributed to approximately 25% of the lost weight, a proportion that is broadly within the range anticipated for meaningful weight loss induced by several mechanisms, including diet and bariatric surgery [62]. Systematic reviews of the existing limited data on body composition with tirzepatide are broadly in agreement with this approximate 25% contribution from LBM loss [64].

In direct comparison with selective GLP-1 RAs, the profile is still developing. Head-to-head trials like SURPASS-2 showed that tirzepatide (all doses) caused greater absolute weight loss compared to semaglutide 1 mg [62]. While this would suggest a likelihood of greater absolute LBM loss with tirzepatide, direct comparative data on body composition changes from this trial are limited. A network meta-analysis suggested that the higher doses of tirzepatide (15 mg), while ideal for adipose loss, may be one of the worst agents to preserve lean mass when compared to some other GLP-1 RAs like liraglutide [66]. These indirect comparisons should be interpreted cautiously due to the heterogeneity among trials. Interpretative comparisons are now possible through the results of SURMOUNT-5, where tirzepatide emerged as superior to semaglutide 2.4 mg regarding weight loss efficacy in adult patients with obesity [67]. Despite the absolute loss in LBM, studies reveal that tirzepatide therapy enhances the overall quality of body composition, manifest through a significant decrease in percentage body fat and a relative rise in the proportion of lean mass as a percentage of total body weight.

### 6.3. Effects on Muscle Function and Risk of Sarcopenia

Definitive evidence on the effect of tirzepatide on direct measures of muscle strength, physical function, or risk of sarcopenia is lacking [64]. The substantial absolute LBM loss observed, particularly at higher dosing, theoretically raises concerns about potential negative effects on muscle function and the risk of sarcopenia, as with the concern described for high-potency selective GLP-1 RAs [47]. This would be of particular concern in older patients or those with baseline frailty or low muscle mass. Reviews have speculated that the dual action of GIP/GLP-1 agonism will have lean mass sparing advantages over GLP-1 agonism, either through an action of GIP on adipose tissue metabolism or through indirect effects of improved insulin sensitivity [68]. Clinical evidence to illustrate a superior muscle-sparing effect or functional benefit is as yet unavailable.

Post-hoc analyses of the SURPASS trials in ≥65-year-old participants demonstrated that tirzepatide induced clinically relevant glycemic improvements and dose-related weight loss, even among participants without baseline obesity (BMI < 30 kg/m^2^), with an acceptable safety profile on hypoglycemia. These analyses did not, however, report specifically on muscle function or sarcopenia endpoints. In the elderly, non-obese population, higher rates of discontinuation owing to adverse events were observed [69].

Tirzepatide represents a significant advancement in achieving substantial weight loss pharmacologically, offering profound benefits for fat mass reduction, including visceral and liver fat, alongside superior glycemic control. This enhanced efficacy, however, is accompanied by significant absolute reductions in LBM. While the proportion of lean mass loss appears comparable to other potent weight loss interventions and leads to improved overall body composition quality (higher % LBM), the sheer magnitude of absolute LBM reduction necessitates a heightened focus on musculoskeletal health. The lack of data on functional outcomes and sarcopenia risk in the context of tirzepatide therapy is still to be discussed. Clinical application, hence, requires not only celebrating the fat loss but also putting into place concurrent strategies, which generally entail structured resistance exercise and sufficient protein intake, to minimize lean mass loss and help maintain muscle function, so that the vascular benefits translate to functional well-being in the long run.

## 7. Comparative Analysis

Since 2005, the development of antidiabetic treatments has offered clinicians a wide range of options, extending beyond basic glycemic control to incorporate the aspects of weight control and cardiorenal protection. The latest international recommendations by the International Diabetes Federation underscore that everyone with T2DM is at risk of the development of metabolic dysfunction-associated steatosis liver disease (MASLD), a condition highly linked to obesity and intermediate hyperglycemia. No antidiabetic agent is yet approved for the use in MASLD, but several agents—such as GLP-1 receptor agonists (semaglutide, liraglutide, tirzepatide), pioglitazone, and SGLT2 inhibitors—have been found to be effective in reducing liver steatosis or the histologic features of MASH. These changes in ectopic fat distribution also underscore the relevance of tailoring antidiabetic treatment according to the patient’s cardiometabolic risk profile and comorbidities [2].

### Direct Comparisons and Relative Positioning (Table 1)

- Weight/Fat Loss: Insulin and sulfonylureas generally cause weight gain, while metformin is neutral or modestly decreases weight [6]. SGLT2 inhibitors cause modest weight/fat loss [38]. GLP-1 RAs cause considerable weight/fat loss, and tirzepatide causes more weight/fat loss than GLP-1 RAs (semaglutide 1 mg) [62].
- Lean Mass Loss: Insulin may increase LBM absolutely but alternatively does so with greater fat gain [20]. The effect of metformin is in contention: neutral or protective in T2D [14]. However, it impairs exercise gains in healthy elderly [16]. SGLT2 inhibitors have been consistently shown to cause modest LBM/SMM loss in absolute terms in meta-analyses [38]. GLP-1 RAs bring about the most pronounced absolute LBM reduction, proportional to the scale of weight loss [41]. Given the greater total weight loss, tirzepatide causes great absolute LBM loss, with some network meta-analyses also suggesting less LBM preservation compared to liraglutide but greater fat loss [62,63,64,65,66]. However, direct head-to-head comparisons considering bias/confounding effects are awaited.
- Sarcopenia Risk/Muscle Function: Negative impacts from insulin contributed by fat gain and lack of functional benefits [29]. Metformin has shown protective interactions observed mostly in T2D but is incompatible with exercise hypertrophy [16]. SGLT2 inhibitors cause more consistent concern, considering the meta-analytical evidence for LBM/SMM loss and possible strength reduction [45]. GLP-1 RAs and tirzepatide bring about the loss of LBM and cause theoretical concerns but no functional data; this loss may be adaptive, and hence it is wise to monitor and mitigate [64].

**Table 1:** Comparative summary table of antidiabetic drugs and their effects on body composition (↓ = decreases).

Feature	Metformin [10,70]	Insulin [71]	GLP-1 Ras [55,56,72]	SGLT2 Inhibitors [42,73]	Tirzepatide [62,64,66,74]
Weight Change	Neutral/Modest loss	Gain	Significant loss	Modest loss	Superior loss
Fat Mass Change	Decrease	Increase	Significant decrease	Decrease	Superior decrease
Visceral Fat Change	Decrease (some evidence)	Increase (potential)	Significant decrease	Decrease	Significant decrease
Lean Mass Change	Neutral/Blunts gain with exercise	Increase (along with fat)	Significant decrease	Significant decrease	Significant decrease
Lean Mass (% Total Loss)	Variable	Variable/Low	~20–50%	~20–50%	~25%
Muscle Function/Strength	Blunted by exercise	No improvement/Worse?	Limited data/Concern	Reduced (Meta-analysis)	Limited data/Concern
Sarcopenia Risk	Lower risk	Complex/Potential risk	Concern/Monitor	Concern/Monitor	Concern/Monitor
Key Mechanisms	AMPK activation, ↓ Inflammation	Anabolism, Fat storage	Appetite suppression, ↓ Gastric emptying	Glycosuria, Caloric loss	Dual incretin agonism, Appetite suppression

Future perspectives include retatrutide, a tri-agonist (GIP/GLP-1/glucagon), that has shown weight loss of up to 24.2% in phase 2 trials. Regarding the quality and the preservation of lean mass, retatrutide’s unique component—glucagon—targets fat oxidation, potentially offering superior body composition outcomes compared to existing therapies [74,75]. There are also trials analyzing tirzepatide combined with a myostatin inhibitor (apitegromab), or semaglutide with the SARM enobosarm, that showed a strong shift in weight loss composition [76,77]. The subjects achieved up to 85–99% fat loss with almost no muscle loss.

## 8. Discussions

The journey of antidiabetic drug development has led to therapies with significant actions beyond glucose control. The considerable weight loss with GLP-1 RAs, SGLT2 inhibitors, and especially dual analog GLP-1/GIP comes with the attendant issue of LBM loss. Although this loss is consistent with fat loss and perhaps adaptive, the LBM decrease is consistent, especially with SGLT2 inhibitors, and its long-term functional effect is unclear. This is a knowledge deficit area, requiring a shift in clinical practice to include active surveillance of muscle status (quantity and function) and integration of muscle-sparing lifestyle interventions with these effective metabolic therapies.

Much of the presented and considered evidence suggests incorporating body composition, particularly muscle health, into the routine management of adult T2D patients. Having only concentrated on glycemic control is not sufficient, given the high prevalence and negative sequelae of sarcopenia and SO in this population. Thus, the selection of an antidiabetic drug should include a careful appraisal of its possible effects on muscle mass and function and its effect on glucose, weight, and cardiorenal risk.

Given the clinical variability of T2D, choosing an antidiabetic medication based on individual features such as BMI, body composition, and glycemic status can result in the most optimal metabolic and functional outcomes. Treatments that preserve or increase lean body mass, such as insulin, may be most effective in people with a low or normal BMI and possible sarcopenia. Treatments for excess adiposity in weight reduction include SGLT2 inhibitors, GLP-1 receptor agonists, and tirzepatide, albeit their impact on lean mass must be considered and decreased. If glycemic control is poor, a combination of powerful glucose-lowering medications and muscle-sparing methods may be the most effective treatment regimen. A patient-centered, composition-sensitive strategy to long-term diabetes therapy could improve outcomes beyond glucose metrics.

Skeletal muscle plays a significant role in insulin-mediated glucose clearance, accounting for over 80% of postprandial glucose uptake. It is controlled by a complicated signaling cascade that includes insulin receptor substrate (IRS)-1/2, phosphoinositide 3-kinase (PI3K), Akt, and glucose transporter type 4 (GLUT4). These pathways become disturbed in T2D, specifically through IRS serine phosphorylation and faulty Akt activation, resulting in decreased GLUT4 translocation and glucose absorption. Chronic inflammation, intramyocellular lipid buildup, and mitochondrial dysfunction all impair insulin signaling and worsen muscle insulin resistance. Such molecular changes not only impair glucose management but also promote protein breakdown in muscle via the ubiquitin-proteasome and autophagy-lysosome pathways, culminating in sarcopenia. As a result, medicines that restore or sustain such signaling pathways can be critical for treating both muscle atrophy and insulin resistance in T2D [78].

Apart from pharmacotherapy, rising evidence supports the use of tailored nutritional therapies to improve body composition and metabolic function. Low-carbohydrate diets are among the most effective nutritional therapies because they directly intervene in T2D’s disrupted glucose metabolism by limiting the primary stimulus of postprandial glucose and insulin secretion [79,80,81]. Clinical trials demonstrate that even a very-low carbohydrate diet (ketogenic) is effective and causes rapid fat loss (including visceral fat) [82,83]. Similarly, a Mediterranean-style eating pattern, which emphasizes healthy fats, fiber, and antioxidants, has been linked to improved glucose metabolism and healthier body composition [84,85,86]. High-protein diets are another option, as they promote higher fat loss while preserving muscle mass, which is especially significant in the elderly or those at risk of sarcopenia [87,88]. Time-restricted feeding, for example, has been proven in randomized trials to increase insulin sensitivity and glycemic management, most likely by matching food intake to circadian cycles [89,90,91]. Finally, polyphenol-rich functional foods, such as berries, green tea, or cocoa, have exhibited favorable benefits on insulin sensitivity and inflammation, with some trials even demonstrating decreases in HbA1c [92] (Table 2).

When interpreting the results of body composition analysis in T2D patients, the potential effect of subclinical thyroid function change should be taken into consideration. Recent evidence shows that in the absence of overt clinical thyroid disease, mild alteration in thyroid hormone levels and thyroid morphology parameters could be strongly related to fluctuations in body composition. A study from noted that TSH, free T3, and indices of thyroid volume were independently related to fat mass, lean mass, and percentage body fat changes among euthyroid individuals [93]. These correlations are likely to reflect the thyroid gland’s intrinsic role in basal metabolic rate and tissue metabolism. Therefore, subclinical thyroid alterations, even within normal clinical ranges, may be modifiers or confounders in studies examining the impact of antidiabetic treatment on body composition. This further emphasizes the need to include thyroid-related factors in future clinical trials when fat and muscle mass alterations are being analyzed.

Long-term functional results must be the priority for subsequent studies by creating randomized controlled trials of over 2–3 years’ duration to evaluate GLP-1 RAs, SGLT2 inhibitors, and tirzepatide compared to robust measures of strength, physical performance, frailty, and incident sarcopenia, along with advanced body composition measures such as MRI/CT-based myosteatosis. Strategies for mitigation to counteract LBM loss, particularly through optimized resistance training regimens for age and health status and individually tailored nutritional interventions (protein sufficiency, timing, quality, and micronutrients like vitamin D), must be stringently evaluated. At-risk populations, including adults ≥ 75 years, the frail, those with sarcopenia, and underrepresented ethnic minorities, must be prioritized to detect differential effects and risks. Studies are also required to unveil how these agents influence muscle protein synthesis, mitochondrial integrity, inflammation, and fat infiltration, with particular emphasis on GIP signaling of tirzepatide. Head-to-head trials of tirzepatide and semaglutide 2.4 mg and SGLT2 inhibitors in large samples with functional and compositional endpoints are of the highest priority. The identification of strong biomarkers for muscle quality and impending functional impairment would also guide clinical care. Finally, real-world evidence from large observational populations will be important to validate trial results in diverse populations and establish long-term effects and adherence.

### 8.1. Strength

The present narrative review provides a synthesis of recent evidence (2015–2025) from RCTs, meta-analyses, and observational studies on primary antidiabetic treatments. A key strength is its inclusion of diverse study designs, offering a balanced perspective on the evidence-based medicine so far.

### 8.2. Limitations

There are important limitations and gaps in the current evidence base. Many studies rely on body composition methods with different precisions, which can be influenced by hydration status, which is particularly relevant for SGLT2 inhibitors. Definitions of sarcopenia vary widely, complicating direct comparisons. Most RCTs, especially for newer agents, have relatively short durations (typically ≤ 1–2 years) for assessing long-term body composition changes and functional outcomes [48].

Another key limitation is the variability in nutritional status and dietary adherence among clinical trial participants. Because diet influences body composition and metabolic results, the absence of standardized food control or monitoring creates a possible bias. This inherent unpredictability, which frequently relies on patient compliance and commitment, can affect the degree of changes in lean or fat mass between different trials.

## 9. Conclusions

The management of T2D has reached an era in which therapies now have core metabolic actions regardless of glycemic control, i.e., remarkable weight loss and cardiorenal protection with GLP-1 RAs, SGLT2 inhibitors, and the dual agonist tirzepatide. The progress is huge, but it is accompanied by the critical challenge of managing concomitant body composition changes, i.e., the sustained reduction in LBM with these new medications. While metformin remains a cornerstone with a net benign or positive impact on muscle, and insulin has a balance of potential mass preservation vs. fat gain, the pronounced weight-reducing action of incretin agents and SGLT2 inhibitors necessitates a paradigm shift. The available evidence, as summarized from research since 2005, is that LBM loss, while proportionate to fat loss and possibly adaptive, nonetheless remains a concern for muscle function and the risk of long-term sarcopenia, especially with SGLT2 inhibitors, where meta-analyses pose the risk of possible losses of strength. Resolution of the current knowledge gaps with long-term studies specifically addressing functional outcomes and preservation strategies is a priority. Functional optimization in adults with T2D is clinically addressed through individualized, concerted attention to monitoring muscle health and function along with glycemia and weight management, supplemented by preventive lifestyle interventions—chiefly resistance exercise and adequate protein intake—to capitalize on the therapeutic potential of modern pharmacotherapies without suffering musculoskeletal compromise and eventual functional dependency.

## Figures and Tables

**Table 2:** Summary table of dietary interventions and body composition in T2D (↓ = decreases).

Dietary Intervention	Key Principles	Summary of Effects on Body Composition
Low-Carbohydrate Diet (e.g., <130 g carbs/day) [79,80,81]	Low carb (<130 g/day), higher protein/fat, lowers insulin & glucose.	Short-term: ~5–7% BW loss, ↓ waist. Long-term like other diets if calories matched.
Ketogenic Diet (very low-carb, high-fat) [82,83]	Very low carb (<50 g/day), high-fat (~70%), induces ketosis.	Most effective for rapid fat loss (~5–10% BW in 3–6 mo), ↓ visceral fat.
Mediterranean Diet (moderate-fat, whole-food diet) [84,85,86]	Whole foods, healthy fats (olive oil), moderate carbs/protein.	Moderate, sustained fat loss (~5% BW in 12 mo), ↓ waist, good adherence.
High-Protein Diet (>25% calories from protein) [87,88]	High-protein (>25% kcal), supports satiety & lean mass retention.	Effective fat loss with calorie deficit. Best muscle preservation when combined with exercise.
Intermittent Fasting (e.g., 5:2 diet, ADF, TRF) [89,90,91]	Cyclic calorie restriction (e.g., 5:2, ADF, TRF), induces fasting benefits.	Fat loss equals daily diets if calories matched. ↓ visceral fat, preserves lean mass.
